# First Trimester Microelements and Their Relationships with Pregnancy Outcomes and Complications

**DOI:** 10.3390/nu12041108

**Published:** 2020-04-16

**Authors:** Małgorzata Lewandowska, Barbara Więckowska, Stefan Sajdak, Jan Lubiński

**Affiliations:** 1 Medical Faculty, Lazarski University, 02-662 Warsaw, Poland; 2 Division of Gynecological Surgery, University Hospital, 33 Polna Str., 60-535 Poznan, Poland; ssajdak@ump.edu.pl; 3 Department of Computer Science and Statistics, Poznan University of Medical Sciences, 60-806 Poznan, Poland; barbara.wieckowska@ump.edu.pl; 4 Department of Genetics and Pathology, International Hereditary Cancer Center, Pomeranian Medical University, 71-252 Szczecin, Poland; lubinski@pum.edu.pl

**Keywords:** selenium, antioxidants, microelements, pregnancy, determinants, risk

## Abstract

Microelements involved in the oxidative balance have a significant impact on human health, but their role in pregnancy are poorly studied. We examined the relationships between first trimester levels of selenium (Se), iron (Fe), zinc (Zn), and copper (Cu), as well as maternal characteristics and pregnancy results. The data came from a Polish prospective cohort of women in a single pregnancy without chronic diseases. A group of 563 women who had a complete set of data, including serum microelements in the 10–14th week was examined, and the following were found: 47 deliveries <37th week; 48 cases of birth weight <10th and 64 newborns >90th percentile; 13 intrauterine growth restriction (IUGR) cases; 105 gestational hypertension (GH) and 15 preeclampsia (PE) cases; and 110 gestational diabetes mellitus (GDM) cases. The microelements were quantified using mass spectrometry. The average concentrations (and ranges) of the elements were as follows: Se: 60.75 µg/L (40.91–125.54); Zn: 618.50 µg/L (394.04–3238.90); Cu: 1735.91 µg/L (883.61–3956.76); and Fe: 1018.33 µg/L (217.55–2806.24). In the multivariate logistic regression, we found that an increase in Se of 1 µg/L reduces the risk of GH by 6% (AOR = 0.94; *p* = 0.004), the risk of IUGR by 11% (AOR = 0.89; *p* = 0.013), and the risk of birth <34th week by 7% (but close to the significance) (AOR = 0.93; *p* = 0.061). An increase in Fe of 100 µg/L reduces the risk of PE by 27% (AOR = 0.73; *p* = 0.009). In the multivariable linear regression, we found negative strong associations between prepregnancy BMI, Se (β = −0.130; *p* = 0.002), and Fe (β = −0.164; *p* < 0.0001), but positive associations with Cu (β = 0.320; *p* < 0.000001). The relationships between Se and maternal age (β = 0.167; *p* < 0.0001), Se and smoking (β = −0.106; *p* = 0.011) and Cu, and gestational age from the 10–14th week (β = 0.142; *p* < 0.001) were also found. Secondary education was associated with Zn (β = 0.132; *p* = 0.004) and higher education was associated with Cu (β = −0.102; *p* = 0.023). A higher financial status was associated with Fe (β = 0.195; *p* = 0.005). Other relationships were statistically insignificant. Further research is needed to clarify relationships between first trimester microelements and pregnancy complications. In addition, attention should be paid to lifestyle-related and socioeconomic factors that affect microelement levels.

## 1. Introduction

Epidemiological data indicate that adverse pregnancy outcomes and pregnancy complications, including preterm births, preeclampsia (PE), gestational diabetes, and fetal growth restriction (IUGR), occur in total in up to 25% of first pregnancies [1] and these pregnancy results are an important element of public health. They are associated with an increase in maternal and fetal morbidity and/or mortality [2,3,4,5,6,7,8,9,10] and they may lead to long-term diseases of both the mother and the child, increasing the risk of cardiovascular diseases, obesity, diabetes, and even some cancers, as well as neurodevelopmental disorders in the offspring [7,8,9,10,11]. Early identification of women at risk may be of great importance for improving pregnancy outcomes. Unfortunately, early risk markers of these pregnancy results remain constantly elusive.

Researchers should pay attention to the microelements involved in the oxidative balance, such as selenium (Se), iron (Fe), copper (Cu), and zinc (Zn) [1]. Importantly, antioxidative and/or anti-inflammatory selenoproteins such as glutathione peroxidases (GPx), thioredoxin reductases (Th-Redx), and selenoproteins P, as well as antioxidative Cu,Zn-SOD (superoxide dismutase) are also active in the uterus [12,13,14,15]. The deficiency of these microelements was previously associated with the low activity of these antioxidative systems, which may be the cause of oxidative damage to placental and fetal tissues [12,13,14,15]. Inadequate levels of the microelements can affect maternal health and the development of the fetus, and early pregnancy is a particular exposure period. However, a role of the microelements in pregnancy (especially in early pregnancy), as well as their determinants in pregnant women are poorly studied.

The studies conducted earlier were mainly animal studies or retrospective studies in humans. Abnormal levels of Se, Fe, Zn, and Cu were found in women with preeclampsia or gestational diabetes mellitus (GDM) [16,17,18]. However, these effects may be due to disorders which have already developed, e.g., increased oxidative stress can increase the consumption of antioxidant elements. There are very few prospective studies. In our Polish prospective cohort of women without chronic diseases recruited in the 10–14th week of a single pregnancy, we examined after the end of pregnancy 121 women developing pregnancy-induced hypertension (PIH) vs. 363 matched women remaining normotensive and we found that a higher risk of PIH was associated with lower serum Se levels in the 10–14th week in a statistically significant way [19]. We also found significant but weaker associations between lower Cu or Fe levels and PIH [20,21]. In the literature, links between lower concentrations of selenium in early pregnancy, and a higher risk of preeclampsia [22,23], preterm birth [5], or birth to infants <10th percentile [24,25,26] have been also found. However, the results of other prospective studies are divergent and the research methodologies differ [1,24,27], e.g., Basu et al. examined serum microelements in a small cohort of 151 pregnant women with preexisting diabetes mellitus, including 23 cases of preeclampsia [27]; and Mistry et al. examined a group of 126 adolescents, including 19 women giving birth to infants <10th percentile [24]. However, only one pregnancy complication has been evaluated in these studies. Importantly, the relationships between maternal characteristics and microelement levels can affect the risk being investigated and this requires the assessment of multiple variables simultaneously. The impact of excessive BMI, smoking, or sociodemographic indicators on microelement levels was also found in the literature but these factors were not assessed in multifactorial models after adjusting for many confounding variables [1,19,20,28].

Summing up, associations between first trimester microelements and their determinants or pregnancy results are poorly studied. In a joint group and using multivariable statistic models, we assessed the relationships between serum concentrations of four microelements (Se, Fe, Zn, and Cu) at the end of the first trimester, as well as main pregnancy outcomes and complications (including gestational age at delivery/preterm births, newborn weight, IUGR, gestational diabetes mellitus (GDM), and pregnancy-induced hypertension (PIH)). The relationships between the microelements, and maternal characteristics (prepregnancy body mass index (BMI), smoking, maternal age, parity, gestational age when microelements were measured, socioeconomic, and demographic characteristics) were also assessed.

## 2. Materials and Methods

### 2.1. Study Population and Method

The data for the current study came from a prospective cohort of pregnant women. The original cohort consisted of 912 women. In the original cohort the following were found: 65 (7.1%) cases of preterm birth <37th week; 72 (7.9%) newborns with a birth weight <10th percentile; 99 (10.9%) newborns >90th percentile; 21 (2.3%) IUGR cases; 137 (15.0%) women developing pregnancy-induced hypertension (PIH); and 146 (16.0%) women developing gestational diabetes mellitus (GDM). Blood samples from mothers were taken during recruitment at the 10–14th gestational week and sera were stored at −80 °C.

The study was performed at the university of obstetric-gynecological and neonatological center (University Hospital in Poznan, Poland, with 6–8 thousand births a year). The study was carried out in accordance with the Helsinki Declaration and was approved by the Bioethics Committee of the Medical University of Poznan, Poland (approval number 769/15), and all the participants signed the Informed Consent Form. The cohort was recruited in 2015–2016; pregnancy results were collected in 2016–2017, and the analysis of data was conducted in 2017–2019.

The criteria for inclusion in this cohort were: Polish Caucasian women of descent from one region (Wielkopolska); aged 18–45 years (at conception); in the 10–14th week of a single pregnancy without aneuploidy; with delivery of a phenotypically normal child ≥ 25th week of pregnancy.

The criteria for exclusion in this cohort were: Chronic diseases (apart from being in excessive body mass index), including preexisting diabetes mellitus, hypertension, immunological and inflammatory diseases, thromboembolism, and kidney or liver diseases.

The participants were recruited from pregnant women taking laboratory tests. Information about the study was available to everyone in the hospital laboratory. Participation in the study was proposed to each of 1300 women who declared that they meet the inclusion criteria (in the period of 12 months in 2015–2016). Participation in the study was voluntary. Until this analysis, missing data concerned 388 (29.9%) participants, including women who did not meet the inclusion criteria after the end of pregnancy and women whose data were incomplete.

Data was collected from a questionnaire during recruitment (in the 10–14th week) and the women filled it out themselves (but in the presence of midwives). The information such as maternal age, weight and height, parity (number of deliveries), history of pregnancy, gynecological histories, concurrent diseases, demographic and socioeconomic characteristics, supplements and medications, family history of pregnancy and chronic diseases, smoking, and consumption of alcohol were collected (all women declared no alcohol in pregnancy). Blood samples from mothers were taken during recruitment from fasting women (women were asked to fast a minimum of 4 h prior to blood collection). Sera were stored at −80 °C.

Height and prepregnancy weight were self-reported. The mother’s height was also measured (in the hospital) before delivery and the values from medical records were included in the study. Prepregnancy body mass index (BMI) for each woman was calculated as a ratio of a weight in kilograms and a height in meters² (kg/m²), and normal BMI was defined as 18.5–24.99 kg/m².

The place of residence included: Village, small town (<50,000 inhabitants), and big city (>50,000 inhabitants). Maternal education categories included: Elementary, vocational, secondary, and higher education; education <12 years included elementary and vocational education; lower education levels included elementary, vocational, and secondary education. The financial status was assessed by means of a 5-point Likert scale for the question “Is your financial situation (in your household) good enough to meet your needs ?”, and maternal responses were classified as follows: Lower financial status (for responses: Definitely NO, rather NO, and hard to say) and higher financial status (for responses: Rather YES and definitely YES).

After the end of pregnancy, results were taken from the hospital medical records. Pregnancy outcomes (including birth weight, intrauterine growth restriction, gestational age at birth, fetal sex, APGAR results) and pregnancy complications (including pregnancy-induced hypertension and gestational diabetes mellitus) have been recorded.

The aim of the current analysis was to assess the relationships between levels of maternal microelements measured in serum samples from the 10–14 gestational week, and main pregnancy results and maternal characteristics. In the current analysis, a group of 563 women who had a complete set of data, including concentrations of the microelements in the 10–14th week was examined. All data came from our original (primary) cohort of pregnant women (n = 912). In the current study, most of the data (together with the concentrations of the elements) came from our previous analysis, in which we examined women who developed pregnancy-induced hypertension and their matched normotensive controls (n = 480); cases and controls were matched in terms of: Mother’s age (±2 years), prepregnancy BMI (±10%), and smoking [19]. Part of the data (n = 83) came from a small pilot analyses conducted among randomly selected normotensive women. Microelements were measured in serum samples after determining the subgroups of women studied (after the end of pregnancy).

In the group of 563 participants the following were found: 47 (8.4%) cases of preterm birth <37th week (vs. 516 childbirth ≥37th week); 48 (8.5%) newborns with a birth weight <10th percentile, and 64 (11.4%) newborns >90th percentile (vs. 451 newborns between the 10–90th percentile); 13 (2.3%) IUGR cases; 120 (21.3%) women developing pregnancy induced hypertension (PIH) (vs. 443 women remaining normotensive); and 110 (19.5%) women developing gestational diabetes mellitus (GDM) (vs. 453 women without GDM). The frequency of the pregnancy results in the study group was close to the frequency in the originally cohort [1,2,5,29,30,31].

### 2.2. Pregnancy Outcomes and Pregnancy Complications

Pregnancy outcomes and complications were taken from the hospital medical records.

The gestational age was estimated based on fetal first trimester ultrasound measurements. Crown-rump length (CRL) measurements were used for pregnancy dating in the 10–13th week of gestation. Above week 14th, other measurements are also considered, as well as combinations thereof.

The birth weight was assessed after birth by means of an electronic scale (in grams). For the assessment of categories of birth weight (<10th and >90th percentile), data from a fetal biometry for the Polish population for the gender of the newborn and for gestational age was used [26]. A diagnosis of intrauterine growth restriction (IUGR) was determined in the utero (via an ultrasound).

Pregnancy-induced hypertension (PIH) was defined in accordance with the national guidelines (2015) and the new definition [2], as “arterial pressure equal to and higher than 140/90 mmHg (on two occasions, at least 4 h apart, in a sitting position, with an oscillometric device) developed de novo after the 20th week of pregnancy, receding up to 12 weeks after delivery.” PIH includes two (main) forms. “Gestational hypertension (GH) was diagnosed if no other disturbance was found; preeclampsia (PE) was diagnosed when any of the following appeared de novo: Proteinuria (≥300 mg/day or ≥0.3 g/L; protein/creatinine ratio ≥0.3; 1+ in the strip test); thrombocytopenia <100 G/L; worsening of renal function; damage to the liver function; pulmonary edema; symptoms from the central nervous system; blurred vision. Intrauterine growth restriction (IUGR) was not one of the criteria of diagnosis”. In this cohort, only proteinuria (≥0.3 g/L) was found in all cases of preeclampsia.

Gestational diabetes mellitus (GDM) was defined in accordance with the national guidelines and the International Association of Diabetes and Pregnancy Study Groups [32]; it was diagnosed based on a 2-h 75 g oral glucose tolerance test (OGTT) after an overnight fast, conducted between the 24th and 28th gestational weeks. Gestational diabetes GDM-1 was diagnosed when modification of the diet was sufficient to control glucose levels. When additional insulin therapy was required, gestational diabetes GDM-2 was diagnosed.

### 2.3. Determination of Microelements

The concentrations of the microelements (Se, Fe, Zn, and Cu) in serum samples were measured. Blood samples from mothers were taken in the 10–14th week of pregnancy (at recruitment) from participants who reported fasting. Samples of venous blood were collected using serum in Z/7.5 mL tubes (Sarstedt Monovette system; Sarstedt, Nümbrecht, Germany).

The tubes were incubated to clot (at room temperature for at least 30 min and no longer than 2 h) and next, samples were centrifuged (at 1300 G for 12 min). Next, sera were stored at −80 °C (until analysis, after the end of pregnancy).

Before determination of the microelements, sera were thawed and centrifuged (at 5000 G for 5 min). The microelement concentrations were determined by means of the Inductively Coupled Plasma Mass Spectrometer: NexION 350D (PerkinElmer, Shelton, CT, USA). The spectrometer was tuned to achieve the manufacturers’ criteria, before an analytical run. Methane was used as a reaction gas.

The instrument was calibrated using an external calibration technique. Calibration standards were prepared from 10 µg/mL of Multi-Element Calibration Standard 3 (PerkinElmer, Shelton, CT, USA) by diluting with a blank reagent to the final concentration of 0.1, 0.5, 1.0, 2.0, 5.0, and 10 µg/L (for Se measurement); 1, 5, 10, and 50 µg/L (for Fe measurement); 1, 5, and 10 µg/L (for Cu measurement); and 1, 5, and 10 µg/L (for Zn measurement). Correlation coefficients for calibration curves were always greater than 0.999.

The analysis protocol assumed a 100-fold dilution of serum in the blank reagent. The blank reagent consisted of 10 mL of 65% Suprapur Grade nitric acid (Merck, Darmstadt, Germany), 0.20 mL of Triton X-100 (PerkinElmer, Shelton, CT, USA) filled to the mark of a 1-L flask with >18 MΩ/cm^2^ deionized water (Merck Millipore, Burlington, MA, USA). Germanium isotope (Ge74) was set as an internal standard.

The accuracy and precision of measurements were tested using a certified reference material (CRM), Clincheck Plasmonorm Serum Trace Elements Level 1 (Recipe, Munich, Germany). Additionally, internal quality control samples were measured during the analysis. General precision was lower than 5% relative standard deviation (RSD). The final concentration included a dilution factor and coefficient which was the mean value of two flanking certified reference material concentrations divided by the mean concentration determined by the manufacturer of CRM [19,20,26,33]. Additional information will be provided on request.

### 2.4. Statistical Analyses

Statistical analyses were performed using the Statistica 13 software. Before statistical analyses, the Shapiro–Wilk test was used to assess the normality of the data distribution. Data distributions were not normal and the Mann–Whitney U test was used to compare microelement medians between pregnancy results or between different categories of maternal characteristics (*p*-value < 0.05 was considered statistically significant). The medians and interquartile ranges (25%–75%) or average microelement concentrations (and standard deviation (SD)) were presented.

The impact of microelement levels (as independent variables) on binary pregnancy results (PIH and GDM and their forms, IUGR and preterm births <37th and <34th week) was assessed in the logistic regression. Adjusted odds ratios (AOR) (and 95% confident intervals (CI)) of pregnancy results for microelement concentrations as continuous variables were calculated in the multivariate logistic regression, and *p*-value was calculated using the Wald test (*p*-value < 0.05 was considered to be significant).

The impact of microelement levels (as independent variables) on gestational age at delivery (as a continuous dependent variable) was also assessed in the multivariable linear regression. The inverse analysis for the impact of binary pregnancy results on the microelement levels is additionally presented in the supplement. One can examine the impact of binary variables on the level of biomarker in the multivariable linear regression, which is possible in the multifactorial model that we used (presence or absence of the disease significantly differentiates the marker level).

The impact of maternal characteristics (as independent variables) on microelement concentrations (as dependent variables) were also evaluated in the multivariable linear regression.

The results of linear regression are presented using the regression coefficient (Beta), the standardized regression coefficient (β), the level of significance (*p*-value < 0.05 was considered statistically significant), and the determination coefficient (R²), which tells what part of the variability of the dependent variable is explained (predicted) by the regression model. Values of 95% confidence interval (95% CI) and adjusted R² (adj R²), which take into account the number of variables in the model were also calculated (data not shown).

The relationships between microelement concentrations and maternal characteristics were evaluated after adjustment for maternal age, prepregnancy BMI, and gestational age at recruitment (variables previously identified as significantly affecting microelement levels in the univariable linear regression). However, the assessed independent variable has always been excluded from the confounding variables. These results were also sustained after PIH was included in the confounding variables (data not shown).

The relationships between microelement levels and PIH, GDM (and their forms), and IUGR, were evaluated after adjusting for maternal age, prepregnancy BMI, gestational age at recruitment, prior PIH, assisted reproductive technology, smoking at recruitment, parity, and education <12 years. The results for preterm birth risk (<37th and <34th week) were obtained after adjusting for maternal age, prepregnancy BMI, gestational age at recruitment, preeclampsia, delivery by caesarean section, parity, fetal sex, and smoking; the results were not sustained in models with premature rupture of membranes (PROM).

## 3. Results

General characteristics of participants (n = 563) are presented in Table 1. The average age was 34.8 years (range 18–45 years), and the average gestational age during recruitment and measurement of microelement concentrations was 12.1 weeks (range 10–14th week). 

The average prepregnancy BMI was 24.9 kg/m² (range 16.5–42.9 kg/m²), and the average gestational age at birth was 38.5 weeks (range 25–42th week). In the whole group (where important risk factors for adverse pregnancy outcomes have been excluded), 15 (2.7%) cases of preeclampsia (PE) and 20 (3.6%) cases of gestational diabetes mellitus with insulin therapy (GDM-2) were found (Table 1).

The relationships between maternal features and the microelements are presented in Table 2, Table 3 and Table 4 and Table S1. The average microelement concentrations (and SD) were presented in Table 2, but medians were compared between subgroups (Table S1). Primiparous had a statistically significantly higher Zn levels compared to multiparous.

Statistically significant relationships between lower selenium (Se) levels and several maternal characteristics (prepregnancy BMI ≥25 kg/m², smoking, maternal age <35 years, lower education level, and lower financial status level) were found. Significant relationships between lower Fe or higher Cu levels, and prepregnancy BMI ≥25 kg/m² were also found. Significant relationships between levels of Fe, Zn, and Cu, and education and financial levels were found.

The impact of maternal features (as independent variables) on microelements in the multivariable model is presented in Table 3. In the multivariable linear regression, Se levels were associated positively with maternal age (*p* < 0.0001), but the women were aged 18–45. Selenium (Se) levels were also associated negatively with smoking (*p* = 0.011). Copper (Cu) levels were associated positively with the gestational age at measurement of microelements (from the 10–14th week) (*p* < 0.001). Prepregnancy BMI was associated negatively with Se (*p* = 0.002) and Fe levels (*p* < 0.0001) but positively with Cu levels (*p* < 0.000001).

The impact of sociodemographic indicators (as independent variables) on microelements in the multivariable model is presented in Table 4. In the multivariable linear regression, zinc (Zn) was associated positively with secondary education (*p* = 0.004), and Cu was associated negatively with higher education (*p* = 0.023). Iron (Fe) was associated positively with a higher financial status (*p* = 0.005).

The relationships between microelements and pregnancy results are presented in Table 5 and Table 6 and Table S2.

The average microelement concentrations (and SD) are presented in Table 5, but medians were compared (Table S2). Women developing pregnancy-induced hypertension (PIH) had significantly lower Se levels compared to the normotensive women (*p* < 0.001). Other relationships were statistically significant but weaker: Lower Se levels in women delivering in <34th compared to deliveries ≥34th week (*p* = 0.043); lower Se levels in IUGR cases compared to controls (*p* = 0.026); lower Fe levels in PIH cases compared to normotensive women (*p* = 0.014); and lower Fe levels in gestational diabetes mellitus (GDM) cases compared to women without GDM (*p* = 0.039). Importantly, lower levels of maternal Se, Fe, and Cu were found simultaneously for birth weight <10th and >90th percentile compared to their controls.

The impact of microelements (as independent variables) on binary pregnancy results in the multivariate model is presented in Table 6. After adjusting for many risk-related variables in the multivariate logistic regression, we found that an increase in Se concentrations of 1 µg/L reduces the risk of pregnancy-induced hypertension (PIH) and isolated gestational hypertension (GH) by 5% (*p* = 0.011) and 6% (*p* = 0.004), respectively. An increase in Se concentrations of 1 µg/L reduces the risk of IUGR by 11% (*p* = 0.013), and the results for preterm birth < 34th week were close to the significance (*p* = 0.061). Among other microelements: An increase in Fe of 100 µg/L reduces the risk of preeclampsia by 27% (*p* = 0.009). The adjusted relationships between microelements and gestational diabetes (GDM) were statistically insignificant, but an increase in Fe of 100 µg/L reduces the risk of GDM-1 by 8% and close to the significance (*p* = 0.056).

The same relationships were calculated in the multivariable linear regression (Table S3) and adjusted results were similar: For the negative relationships between Se levels and PIH (β = −0.178; *p* < 0.001), GH (β = −0.175; *p* <0.001), and IUGR (β = −0.095; *p* = 0.022). The adjusted relationships between Se and gestational age at birth were positive (β = 0.083; *p* = 0.044) but the results were not sustained in the models with premature rupture of membranes (PROM). The relationships between Fe and preeclampsia also were significant and negative (β = −0.094; *p* = 0.026). Additionally (Table S3), we found that levels of Cu were associated negatively with PIH (β = −0.098; *p* = 0.030). Negative relationships between Fe and GDM-1 were insignificant (β = −0.051; *p* = 0.229), but positive relationships between Cu and GDM-1 were close to the significance (β = 0.076; *p* = 0.057).

The short characteristics of mothers in the case groups (PIH or preterm birth) and their controls are presented in Table S4.

## 4. Discussion

In this study, heathy women with a single pregnancy were recruited in the 10–14th week. After the end of pregnancy, outcomes were recorded. For 563 women, the microelement measurements (in serum samples collected in the 10–14th week) were available.

In the analyses of pregnancy results, strong statistically significant lower levels of Se in women developing pregnancy-induced hypertension (PIH) (including isolated gestational hypertension (GH)) compared to the normotensive women were found (*p* < 0.001), and these relationships were sustained in the multivariable models (Table 5 and Table 6 and Table S3). Other relationships were statistically significant but weaker: Lower Se levels in women delivering in <34th compared to deliveries ≥34th week; lower Se levels in IUGR cases compared to controls; lower Fe levels in PIH/preeclampsia cases compared to normotensive women (Table 5 and Table 6 and Table S3). 

The relationships between lower Fe levels and gestational diabetes mellitus (GDM) compared to women without GDM (Table 5) were not sustained in the multivariable models but were close to the significance (Table 6). After adjusting for many risk-related variables, positive relationships between Cu and GDM-1 also were close to the significance. Other relationships were statistically insignificant in the models we used.

At the same time, the determinants of microelement levels in early pregnancy were: Prepregnancy BMI (for Se, Fe, and Cu), smoking (for Se), maternal age (for Se), and gestational age between 10–14th week (for Cu), as well as lower education (for Zn and Cu) and financial status (for Fe).

In the literature, there is little prospective research on this topic.

The most frequently studied form of pregnancy-induced hypertension (PIH) is preeclampsia. Among prospective studies, the development of preeclampsia was associated with lower Se levels in a study by Ghaemi et al. [22] and Rayman et al. [22,23]. The prospective role of lower Cu levels in predicting preeclampsia were found by Basu et al. and Ugwuja et al. [27,34]. In the very small pilot prospective study, Tande et al. found statistically lower maternal first trimester serum iron (Fe) levels in the women who subsequently developed gestational hypertension, compared to controls [35]. In our previous case-control analyses in matched groups, women in the lowest (Q1) quartile of Se concentrations compared to the women in the highest (Q4) quartile had a 15 times higher PIH risk, in the subgroup of women with the normal prepregnancy BMI [19]. Women in lower quartiles (Q1–Q2) of Fe or Cu levels (compared to the highest Q4 quartiles) also had a higher risk of PE or PIH/GH respectively, but the results were weaker [20,21].

Preterm birth was associated with lower Se levels in early pregnancy in a study by Rayman et al. [5]. Retrospective studies also showed lower serum Se levels in pregnant women undergoing preterm birth compared to the term group [36,37]. Lower Se levels were also found in women delivering newborns with IUGR [12].

Gestational diabetes mellitus (GDM) was not prospectively associated with first trimester maternal Se, Cu, Zn, and Fe levels [1,28]. In women with GDM (in retrospective studies), the relationships between higher levels of plasma Cu and GDM were found [17,18]. Retrospective studies indicate that there are discrepancies in the relationships between Fe, Se, and Zn, and GDM risk [38,39,40,41].

The meta-analyses indicate that the relationships between lower levels of micronutrients and SGA (birth weight <10th percentile) are inconsistent and weak [42]. Among the few prospective studies, relationships between lower levels of Se and SGA were found by Mistry et al. and Bogden et al. [24,25]. Our previous study in matched groups also showed the relationships between lower Se levels in early pregnancy and a higher risk of SGA [26]. However, results of other studies are divergent [1,28]. Choi et al. found no relationship between lower Se levels and a higher SGA risk (after adjusting for all the trimesters of pregnancy); but they found that lower Se levels were associated with a higher birth weight [28]. Our earlier analysis also showed the relationships between lower Se levels in early pregnancy, and a higher LGA risk (large-for-gestational age birth weight) [33].

In our group of 563 participants (in the current analysis), the frequency of the pregnancy outcomes (8.4% of the deliveries were <37th week; 8.5% of the women gave birth to newborn <10th percentile, and 11.4% gave birth to newborn >90th percentile) was close to the frequency in other Polish studies [43,44]. In our analysis, the frequency of main complications (21.3% of the women have developed PIH and 19.5% have developed GDM) was about 2–4 times higher compared to the frequency in several Polish studies [43,45,46]. At the same time, among our participants, 40.7% of the women were primiparous, 39.1% had prepregnancy BMI ≥25 kg/m², and 63.6% were ≥35 years. Firstly, such a “structure” of our group may result from the fact that our study was conducted in the third-degree reference center, to which women with risk factors apply. These women with risk factors are also more willing to participate in additional research. Secondly, our group included participants from our two previous analyses based on data from the common primary cohort, but most of the data (n = 480) came from the analysis of PIH cases and their matched normotensive controls. Importantly, we also obtained our results after correcting for PIH cases (data not shown).

Our prospective cohort was recruited with an exclusion of multiple pregnancy and chronic diseases such as preexisting hypertension or diabetes mellitus, immunological and inflammatory diseases, thromboembolism, and kidney or liver diseases (important risk factors of preeclampsia). The low frequency of preeclampsia (2.7%) in our study is consistent with the literature data and our methodology. According to the latest FIGO report from 2019 (The International Federation of Gynecology and Obstetrics), preeclampsia affects 2%−5% of pregnant women [47]. In the current analysis, the number of IUGR cases (n = 13) and the number of preeclampsia cases (n = 15) was small and the results for these cases should be evaluated with caution and treated only as a pilot study. 

The mechanisms of the results identified are not fully explained, but the relationships between lower microelement levels and oxidative stress intensification are taken into account. Selenium (Se), zinc (Zn), copper (Cu), and iron (Fe) are involved in many biochemical processes and show antioxidant and anti-inflammatory properties, and their deficiency (or excess) is associated with an increase in oxidative stress and inflammation [12,13,14,15,48].

Oxidative stress and inflammation have been shown to be important in the pathogenesis of adverse pregnancy results [3,12,13]. Pregnancy induced hypertension is characterized by an increase de novo in blood pressure after the 20th week of pregnancy, however, evidence available in the literature suggests that adverse pregnancy outcomes and complications may be due to placental dysfunction in early pregnancy [12,49]. Disorders of trophoblast invasion (during placental development before the 20th week) result in the development of high-resistance placental circulation, reduction of blood flow in the placental circulation, and hypoxia, as well as an increase in levels of reactive oxygen species (ROS) and an increase in oxidative stress. It affects the immune system and apoptosis and increases inflammatory response. Adequate levels of antioxidative and/or anti-inflammatory selenoproteins (such as glutathione peroxidases (GPx), thioredoxin reductases (Th-Redx), and selenoproteins P) are needed to protect placental, fetal, and maternal tissue against oxidative stress and oxidative damage [12]. Literature data show that lower levels of Se were associated with a lower activity of these selenoproteins [12]. Increased oxidative stress and many other processes in the placenta and systemic circulation system result in the damage of the vascular endothelium and disturbance of endothelial homeostasis [12]. The activation of many markers (e.g., oxidative stress markers, inflammatory cytokines, von Willebrand factor, endothelin-1, fibronectin, etc.) and deficiency of vasodilators (as nitric oxide and prostacyclin) result in blood pressure increase [11,12,13,14,15,48,49].

In mechanisms of preterm birth development, the relationships between lower Se levels and oxidative stress and inflammation intensification and other factors, such as weakening of the immune protection and increased risk of infection, are taken into account [5,50]. Placental insufficiency is also the cause of IUGR [12].

However, it remains unexplained and poorly investigated as to what is the cause of abnormal trophoblast invasion in the first half of pregnancy. Genetic, environmental, and metabolic factors are considered, including oxidative stress and inflammation, as the primary cause [49]. A deficiency of selenium and inadequate activity of antioxidative systems in the uterus can cause disorders of the trophoblast cell function. Moreover, other activities of this element are taken into consideration. Deficiency of their antioxidant and anti-inflammatory effects in early pregnancy may be associated with the development of adverse pregnancy results [12,13].

Although the diet is a basic determinant of microelement levels in the body [51], various factors, e.g., obesity, smoking, alcohol, or older age, can change their levels in tissues, as several studies in the general population have shown [52,53], and these factors have been associated with increased oxidative stress and inflammation [52,54,55,56,57,58]. However, the number of studies on pregnant women is limited [1,28,54]. Importantly, lifestyle indicators (such as obesity, smoking, or alcohol consumption) and lower socioeconomic factors (lower education or financial levels) increase the risk of adverse pregnancy outcomes [2].

Obesity has been associated with lower levels of Se, Fe, and Zn in the general population [54,55,59,60,61], though there are also discrepancies which is consistent with our negative relationship between maternal prepregnancy BMI and Se or Fe [62]. The statistically and positive relationships between Cu levels and prepregnancy BMI found in our study are also confirmed in the literature and is explained by the increase in Cu-binding ceruloplasmin (acute phase protein) [63].

Smoking is mentioned as a factor that lowers Se levels in the general population [52]. A reduction of Se levels in smokers may result from interactions, e.g., with cadmium accumulated in smokers’ bodies [13,15,64]. Aging is associated with the lowering of Se levels [52]; however in the present study, women aged 18–45 were evaluated and statistically positive relationships between maternal age and Se levels were found. It means that other factors can also affect these correlations, e.g., diet or hormonal factors [65].

The literature confirms the increase in Cu concentrations along with the duration of pregnancy and it is explained by the synthesis of ceruloplasmin (it has not been fully elucidated) [66,67], but it is noteworthy that the gestational age range (when microelements were measured) in our study was narrow (from the 10–14th week). According to the literature, the levels of Se, Zn, and Fe are reduced in pregnancy [12,28,54,67,68].

Socioeconomic indicators, geographical location, seasonality, race and ethnic differences, as well as gender also affect the level of elements [28,52,69]. Lower socioeconomic factors may be associated with an unhealthy lifestyle, including improper diet, obesity, smoking, or noncompliance with medical recommendations, e.g., regarding vitamin supplementation in pregnancy [28]. Different diets at different times of the year can affect seasonal differences in microelement levels [69].

Geographical differences in the content of elements in soil mainly concern selenium and affect the differences in the content of this element in plants and animals, and then in people [70,71]. In developed countries, there is currently no deficiency of zinc, which may be the result of various national dietary interventions [72]. Low levels of selenium are found in some regions of the world [70,71]. In our country, the average Se concentration is 70 µg/L (50−55 µg/L in some regions), and in North America it is 122.4−151.8 µg/L [70,73,74]. Recommendations for the optimal amount of Se in the diet are based on maximizing glutathione peroxidase (GPx) [70,71]. The optimal mean serum concentration of Se (according to the World Health Organization) is 39.5–194.5 µg/L for healthy adults, and 70−90 µg/L was considered to maximize GPx. The average demand (estimated average requirement (EAR)) was estimated at 45 µg/day for people aged 19–50 years. In pregnant women, the recommended dietary allowance (RDA) increases to 60 µg/day [75]. It should also be emphasized that the adverse effects of selenium on health have a “U” curve, therefore supplementation should only be used in people with a known Se deficiency. Optimizing the Se level through a balanced diet seems to be a safe method [70].

In our opinion, the current study has two main implications for clinical practice. Measuring assessed microelements (especially selenium) in early pregnancy can help identify women who have been evaluated as at risk of pregnancy complications and increase care for them; measurement of microelements is simple and available. Importantly, measurement of microelements may suggest the need for changes in diet and in health-promoting behaviors (prepregnancy BMI reduction and smoking cessation). Certain lifestyle modifications should be performed as a matter of course in every pregnant woman and not only based on low levels of microelements. The obtained results deserve further research. Additionally, the possibility of including microelement measurements in early pregnancy (or maybe before pregnancy) can be considered for screening.

### Advantages and Limitations

The main advantage was the evaluation of the prospectively collected group and analysis of microelements in serum samples collected at the end of the first trimester of pregnancy. The advantage of the current study was a fairly large sample size, but further studies on larger samples are advisable. An advantage was simultaneously examining the relationship between the microelements, and the features of mothers and several pregnancy results. Another advantage of this study was the use of multivariable models of statistical analyses.

The limitation of this study may be the lack of analysis of the diet composition, but the concentrations of microelements in the serum were assumed to be a reflection of the consumption of elements (in the diet and possible supplements). We could not explore other ethnic groups because they are few in our country. The exclusion of diseases with elements of inflammation and oxidative stress was good for assessing the effect of “oxidative stress” microelements on the pregnancy results, but reduced the frequency of preeclampsia (PE). The women reported their body weight before pregnancy on their own.

## 5. Conclusions and Future Work

This study shows strong relationships between lower levels of selenium in early pregnancy and development of pregnancy-induced hypertension (PIH), including isolated gestational hypertension (GH). The weaker relationships between lower levels of microelements (Se, Fe, or Cu) and PIH, preeclampsia, preterm birth, IUGR, and gestational diabetes mellitus (GDM) were also found. At the same time, attention should be paid to lifestyle-related factors and lower socioeconomic indicators that affect microelement levels. Our results suggest the need for changes in health-promoting behaviors. Of course, healthy behavior is needed for every pregnant woman, not only to optimize the level of microelements.

The obtained results deserve further research to clarify whether there is a causal relationship and whether the role of selenium in the early prediction of PIH is possible. It is possible, that nutritional interventions in the peri-conceptual period can help in the prevention of this pregnancy complication. It would be interesting to probably reveal differences between certain research groups, such as different racial and ethnic groups, and different geographical locations.

## Figures and Tables

**Table 1:** General characteristics of the whole group.

General Characteristics			
Microelements *	**N**	**Median**	**(25%–75% ranges)**
Selenium (µg/L)	563	60.27	55.08–65.04
Iron (µg/L)	563	993.94	801.20–1208.42
Zinc (µg/L)	563	606.61	548.54–662.89
Copper (µg/L)	563	1727.10	1532.84–1923.11
**Maternal characteristics**		**Mean (±SD) or n**	**Range or (%)**
Maternal age (years)	563	34.8 (4.4)	18–45
Maternal age ≥35 years	563	358	(63.6%)
Number of prior births (n)	563	0.87 (0.91)	0–5
Primiparous	563	229	(40.7%)
Gestational age at recruitment (week)	563	12.1 (0.9)	10–14
Prepregnancy BMI (kg/m^2^)	563	24.9 (4.7)	16.5–42.9
Prepregnancy BMI ≥25 kg/m²	563	220	(39.1%)
Smokers #	563	36	(6.4%)
Lower education levels ##	468	175	(37.4%)
Lower financial status ###	214	83	(38.8%)
**Pregnancy outcomes**		**Mean (±SD) or n**	**Range or (%)**
Fetal sex—Son	563	294	(52.2%)
Gestational age at birth (week)	563	38.5 (2.0)	25–42
Childbirth <37th week	563	47	(8.4%)
Childbirth <34th week	563	17	(3.0%)
Childbirth ≥37th week	563	516	(91.7%)
Birth weight (g)	563	3315.9 (618.2)	495.0–5200.0
Birth weight <10th percentile	563	48	(8.5%)
Birth weight >90th percentile	563	64	(11.4%)
Birth weight between the 10–90th percentile	563	451	(80.1%)
Intrauterine growth restriction (IUGR)	563	13	(2.3%)
Pregnancy-induced hypertension (PIH) *	563	120	(21.3%)
Normotensive controls	563	443	(78.7%)
Gestational diabetes mellitus (GDM) **	563	110	(19.5%)
Women without GDM	563	453	(80.5%)

* Normal ranges for the microelements (for not pregnant women >18 years) according to laboratory data in the region: Zn 680–1070 µg/L; Cu 800–1550 µg/L; Fe 600–1800 µg/L. Normal ranges for Se are estimated at 60–120 µg/L; # smokers during recruitment; ## elementary, vocational, and secondary education; ### in the 5-point Likert scale (lower status included 1–2–3 level); * pregnancy-induced hypertension (PIH) included 105 cases of gestational hypertension (GH) and 15 cases of preeclampsia (PE); ** gestational diabetes mellitus (GDM) included 90 cases with dietary modification (GDM-1) and 20 cases with additional insulin therapy (GDM-2).

**Table 2:** Maternal characteristics and microelement concentrations (in serum from the 10–14th week of pregnancy).

Microelement Concentrations (µg/L) * (Mean ± SD)					
		SELENIUM (Se)	IRON (Fe)	ZINC (Zn)	COPPER (Cu)
Maternal Characteristics	n				
Parity					
Primiparous	229	60.87 (10.15)	1027.89 (338.26)	640.08 (209.67)	1715.10 (229.00)
Multiparous	334	60.67 (7.00)	1011.78 (344.66)	603.69 (84.32)	1750.18 (293.38)
*p*-value		0.328	0.730	0.007	0.065
Maternal age (years)					
Age ≥35	358	61.56 (41.14)	1003.07 (319.75)	614.15 (96.32)	1737.13 (312.94)
Age <35	205	59.34 (40.91)	1044.98 (376.72)	626.08 (212.83)	1733.78 (335.20)
*p*-value **		0.0002	0.468	0.862	0.652
Prepregnancy BMI (kg/m²)					
BMI ≥25	220	59.84 (44.39)	996.95 (335.21)	629.17 (209.65)	1846.21 (357.11)
BMI <25	343	61.34 (40.91)	1051.29 (342.50)	611.65 (92.16)	1665.16 (273.38)
*p*-value **		0.006	0.002	0.893	<0.00001
Smoking at recruitment					
Yes	36	56.79 (40.91)	1053.31 (369.45)	622.20 (89.34)	1718.79 (272.24)
No	527	61.02 (42.68)	1015.94 (340.14)	618.24 (152.86)	1737.08 (324.18)
*p*-value **		0.009	0.612	0.353	0.645
Education					
Higher	293	61.59 (42.68	1023.05 (334.68)	606.79 (87.16)	1699.65 (284.66)
Other (Lower levels)	175	59.62 (40.91)	1030.44 (361.58)	640.13 (227.81)	1798.29 (340.71)
*p*-value **		0.0005	0.720	0.197	0.001
Financial status ***					
Higher status (4–5)	131	61.91 (40.91)	1106.05 (337.95)	614.10 (92.51)	1736.00 (300.54)
Lower status (1–2–3)	83	59.30 (44.85)	933.87 (319.38)	641.96 (311.84)	1799.13 (376.59)
*p*-value **		0.006	0.001	0.610	0.394
Place of residence					
Village	160	60.08 (7.17)	1027.58 (345.65)	629.20 (229.31)	1748.43 (295.50)
Other	402	61.02 (8.87)	1015.55 (340.66)	614.35 (102.14)	1731.30 (331.03)
*p*-value		0.324	0.910	0.767	0.516

* Microelement concentrations were measured in serum samples from the 10–14th gestational week; ** *p*-value was obtained using the Mann–Whitney U test and medians were compared (*p* < 0.05 was considered to be significant); *** financial status in the 5-point Likert scale (lower status included 1–2–3th levels and higher status included 4–5th levels); BMI: Body mass index.

**Table 3:** The adjusted relationships between maternal features and microelement levels.

Impact of Maternal Features on Microelement Levels in the Multivariable Linear Regression #				
	Beta *	β **	*p* ***	R² ** **
**SELENIUM (Se)**				
Maternal age (years)	0.319	0.167	0.00007	0.040
Number of prior births	−0.514	−0.056	0.207	0.043
Prepregnancy BMI (kg/m²)	−0.232	−0.130	0.002	0.040
Gestational age at recruitment (weeks)	0.405	0.041	0.325	0.042
Smoking at recruitment	−3.627	−0.106	0.011	0.051
**IRON (Fe)**				
Maternal age (years)	−3.866	−0.050	0.236	0.029
Number of prior births	−11.635	−0.031	0.458	0.028
Prepregnancy BMI (kg/m²)	−11.871	−0.164	0.00009	0.027
Gestational age at recruitment (weeks)	17.545	0.044	0.296	0.025
Smoking at recruitment	50.478	0.036	0.387	0.028
**ZINC (Zn)**				
Maternal age (years)	−1.579	−0.046	0.275	0.004
Number of prior births	−13.052	−0.080	0.060	0.008
Prepregnancy BMI (kg/m²)	1.421	0.045	0.288	0.002
Gestational age at recruitment (weeks)	−11.634	−0.066	0.118	0.006
Smoking at recruitment	2.417	0.004	0.925	0.002
**COPPER (Cu)**				
Maternal age (years)	−2.741	−0.038	0.346	0.127
Number of prior births	−2.326	−0.007	0.868	0.126
Prepregnancy BMI (kg/m²)	21.760	0.320	<0.000001	0.126
Gestational age at recruitment (weeks)	53.773	0.142	0.0004	0.126
Smoking at recruitment	−34.088	−0.026	0.512	0.126

# Relationships between variables were calculated after adjusting for maternal age, prepregnancy BMI, and gestational age at recruitment (variables identified in the univariable linear regression as significantly affecting microelement levels) but the assessed independent variable has always been excluded from the confounding variables; * Beta: Unstandardized regression coefficient ** β: Standardized regression coefficient; *** *p* < 0.05 was considered to be significant; ** ** R²: Coefficient of determination.

**Table 4:** The adjusted relationships between sociodemographic indicators and microelement levels.

Impact of Sociodemographic Indicators on Microelement Levels in the Multivariable Linear Regression #				
	Beta *	β **	*p* ***	R² ** **
**SELENIUM (Se)**				
Higher education	1.332	0.080	0.082	0.085
Secondary education	−0.826	−0.045	0.316	0.081
Education <12 years	−1.467	−0.057	0.214	0.082
Higher financial status #	1.835	0.114	0.093	0.126
Village	−0.892	−0.048	0.252	0.044
Town <50,000 inhabitants	0.416	0.023	0.583	0.042
City >50,000 inhabitants	0.381	0.022	0.594	0.042
**IRON (Fe)**				
Higher education	−35.44	−0.050	0.288	0.036
Secondary education	61.17	0.078	0.088	0.039
Education <12 years	−41.675	−0.038	0.418	0.035
Higher financial status #	135.790	0.195	0.005	0.091
Village	16.646	0.022	0.601	0.031
Town <50,000 inhabitants	−2.209	−0.003	0.943	0.031
City >50,000 inhabitants	−12.061	−0.017	0.680	0.031
**ZINC (Zn)**				
Higher education	−30.515	−0.095	0.046	0.017
Secondary education	46.800	0.132	0.004	0.026
Education <12 years	−23.800	−0.048	0.313	0.011
Higher financial status #	−28.448	−0.067	0.351	0.004
Village	16.472	0.050	0.242	0.011
Town <50,000 inhabitants	−8.182	−0.025	0.550	0.009
City >50,000 inhabitants	−6.594	−0.022	0.610	0.009
**COPPER (Cu)**				
Higher education	−65.022	−0.102	0.023	0.131
Secondary education	55.496	0.079	0.071	0.127
Education <12 years	40.315	0.041	0.361	0.122
Higher financial status #	−6.586	−0.010	0.884	0.162
Village	−7.255	−0.010	0.798	0.127
Town <50,000 inhabitants	14.082	0.020	0.610	0.127
City >50,000 inhabitants	−6.431	−0.010	0.805	0.127

# Relationships between variables were calculated in the multivariable linear regression after adjusting for maternal age, prepregnancy BMI, and gestational age at recruitment (variables identified in the univariable linear regression as significantly affecting microelement levels); # financial status in the 5-point Likert scale (lower status included 1–2–3th levels and higher status included 4–5th levels); * Beta: Unstandardized regression coefficient ** β: Standardized regression coefficient.; *** *p* < 0.05 was considered to be significant; ** ** R²: Coefficient of determination.

**Table 5:** The microelement concentrations (in the 10–14th week of pregnancy) and pregnancy results.

Microelement Concentrations (µg/L) * (Mean ±SD)					
		SELENIUM (Se)	IRON (Fe)	ZINC (Zn)	COPPER (Cu)
Pregnancy Results	n				
Gestational age at birth					
<34th week	17	56.71 (40.91)	904.82 (270.51)	599.35 (85.06)	1832.31 (393.00)
≥34th week	546	60.88 (41.14)	1021.87 (343.44)	619.09 (151.14)	1732.91 (318.39)
*p*-value **		0.043	0.186	0.388	0.188
Gestational age at birth					
<37th week	47	58.97 (8.20)	925.40 (232.10)	604.76 (75.82)	1751.74 (335.29)
≥37th week	516	60.91 (8.43)	1026.80 (349.09)	619.75 (154.52)	1734.47 (319.89)
*p*-value **		0.148	0.091	0.561	0.556
Birth weight <10′					
<10 percentile	48	59.60 (41.14)	969.28 (301.28)	631.09 (97.19)	1651.58 (268.92)
≥10 percentile	515	60.89 (40.91)	1022.90 (345.31)	617.32 (153.55)	1743.77 (324.46)
*p*-value **		0.303	0.374	0.143	0.031
Birth weight >90′					
>90 percentile	64	59.66 (45.17)	974.11 (295.33)	616.04 (84.85)	1731.86 (301.55)
≤90 percentile	499	60.86 (40.91)	1024.00 (347.23)	618.81 (156.00)	1736.43 (323.61)
*p*-value **		0.296	0.361	0.903	0.853
IUGR					
Yes	13	55.59 7.90	910.08 271.15	636.82 64.06	1577.29 336.24
No	550	60.87 8.40	1020.89 343.14	618.06 151.00	1739.66 319.93
*p*-value		0.026	0.316	0.159	0.113
Pregnancy-induced hypertension (PIH)					
PIH cases	120	57.56 (40.91)	948.86 (333.73)	610.16 (87.95)	1699.48 (299.35)
Normotensive controls	443	61.62 (41.14)	1037.15 (341.97)	620.75 (162.28)	1745.78 (326.15)
*p*-value **		0.000002	0.014	0.976	0.235
Gestational diabetes mellitus (GDM)					
GDM cases	110	61.88 (44.39)	964.03 (313.88)	629.20 (104.66)	1798.27 356.53
Women without GDM	453	60.48 (40.91)	1031.52 (347.36)	615.90 (158.54)	1720.77 310.21
*p*-value **		0.161	0.039	0.084	0.061

* Microelement concentrations were measured in serum samples from the 10–14th gestational week; ** *p*-value was obtained using the nonparametric Mann–Whitney U test (*p* < 0.05 was considered to be significant; IUGR: Intrauterine growth restriction; PIH: Pregnancy-induced hypertension (105 cases of gestational hypertension and 15 cases of preeclampsia); GDM: Gestational diabetes mellitus (90 cases of GDM-1 and 20 cases of GDM-2).

**Table 6:** The adjusted odds ratios of pregnancy results for levels of microelements.

Adjusted Odds Ratios of Pregnancy Results			
	AOR *	(95% CI:)	*p* **
**For selenium (Se) concentrations (for 1 µg/L)**			
Pregnancy-induced hypertension (PIH)	0.95	0.91–0.99	0.011
Isolated gestational hypertension (GH)	0.94	0.90–0.98	0.004
Preeclampsia (PE)	1.03	0.95–1.10	0.490
IUGR	0.89	0.82–0.98	0.013
Gestational Diabetes Mellitus (GDM)	1.01	0.97–1.04	0.748
GDM-1	1.01	0.97–1.04	0.760
GDM-2	1.00	0.92–1.08	0.914
Preterm birth <34 week	0.93	0.86–1.00	0.061
Preterm birth <37 week	0.97	0.92–1.01	0.117
**For iron (Fe) concentrations (for 100 µg/L)**			
Pregnancy-induced hypertension (PIH)	0.93	0.85–1.02	0.115
Isolated gestational hypertension (GH)	0.98	0.90–1.07	0.655
Preeclampsia (PE)	0.73	0.57–0.92	0.009
IUGR	0.89	0.74–1.07	0.222
Gestational Diabetes Mellitus GDM	0.94	0.86–1.01	0.108
GDM-1	0.92	0.84–1.00	0.056
GDM-2	1.04	0.87–1.22	0.687
Preterm birth <34 week	0.96	0.81–1.14	0.644
Preterm birth <37 week	0.94	0.85–1.04	0.258
**For zinc (Zn) concentrations (for 100 µg/L)**			
Pregnancy-induced hypertension (PIH)	0.86	0.67–1.10	0.229
Isolated gestational hypertension (GH)	0.90	0.73–1.11	0.331
Preeclampsia (PE)	0.72	0.37–1.38	0.323
IUGR	1.05	0.83–1.34	0.680
Gestational Diabetes Mellitus GDM	1.00	0.86–1.17	0.984
GDM-1	0.97	0.80–1.18	0.784
GDM-2	1.08	0.85–1.37	0.527
Preterm birth <34 week	0.84	0.48–1.46	0.532
Preterm birth <37 week	0.91	0.66–1.26	0.575
**For copper (Cu) concentrations (for 100 µg/L)**			
Pregnancy-induced hypertension (PIH)	0.93	0.85–1.02	0.138
Isolated gestational hypertension (GH)	0.95	0.86–1.04	0.252
Preeclampsia (PE)	0.95	0.78–1.15	0.597
IUGR	0.83	0.67–1.02	0.075
Gestational Diabetes Mellitus GDM	1.04	0.96–1.13	0.371
GDM-1	1.04	0.96–1.14	0.332
GDM-2	1.01	0.83–1.21	0.952
Preterm birth <34 week	1.08	0.92–1.26	0.336
Preterm birth <37 week	0.99	0.90–1.10	0.901

* AOR: Adjusted odds ratios calculated in the multivariate logistic regression; ** *p*-value obtained using the Wald test (*p* < 0.05 was considered to be significant); the results for risk of IUGR were obtained after adjusting for maternal age, prepregnancy BMI, gestational age at recruitment, smoking, parity, prior PIH, assisted reproductive technology; the results for risk of PIH and GDM were obtained in the same model + education level < 12 years; the results for risk of preterm births (<37th and <34th week) were obtained after adjusting for maternal age, prepregnancy BMI, gestational age at recruitment, smoking, preeclampsia, delivery by caesarean section, parity, and fetal sex (the results were not sustained in models with PROM); CI: Confidence intervals.

## Supplementary Materials

The following are available online at https://www.mdpi.com/2072-6643/12/4/1108/s1, Table S1: Full characteristics of microelement levels (medians and ranges) for various maternal characteristics, Table S2: The adjusted relationships between maternal characteristics and microelement levels after additional adjusting for pregnancy-induced hypertension (PIH), Table S3: Full characteristics of microelement levels (medians and ranges) for pregnancy results, Table S4: Adjusted relationships between pregnancy results and microelements in the multivariable linear regression, Table S5: Short characteristics of mothers in the control and case groups (for PIH and preterm birth).
