# Supplementary material for: First Trimester Microelements and Their Relationships with Pregnancy Outcomes and Complications
Source: Nutrients. 2020 Apr 16;12(4):1108. doi: 10.3390/nu12041108 (PMC7230599; doi:10.3390/nu12041108)
Supplement: Supplementary file 1 [file nutrients-12-01108-s001.pdf]

Table 1. Full characteristics of microelement levels (medians and ranges) for various maternal characteristics.

	n	Mean	SD	Q25	Median	Q75	p **
**SELENIUM (Se) ***							
Multiparous	334	60.67	7.00	55.70	60.33	65.11	0.328
Primiparous	229	60.87	10.15	54.24	59.95	64.47	
Age <35 years	205	59.34	9.08	53.65	58.42	63.65	**0.0002**
Age ≥35 years	358	61.56	7.91	56.25	61.07	65.82	
Prepregnancy BMI <25kg/m²	343	61.33	8.04	55.82	60.91	65.40	**0.006**
Prepregnancy BMI ≥25 kg/m²	220	59.84	8.92	54.18	58.71	64.34	
Smoking at recruitment—No	527	61.02	8.43	55.38	60.41	65.16	**0.009**
Smoking at recruitment —Yes	36	56.79	7.21	53.44	57.46	63.06	
Higher education—No	175	59.62	9.32	53.57	58.20	64.92	**0.0005**
Higher education—Yes	293	61.59	7.22	56.78	60.99	65.73	
Village—No	402	61.02	8.87	55.70	60.25	65.11	0.324
Village—Yes	160	60.08	7.17	54.24	60.23	63.86	
Lower financial status ***	83	59.30	8.91	53.73	58.89	64.43	**0.006**
Higher financial status	131	61.91	6.97	57.12	60.82	66.64	
**IRON (Fe) ***							
Multiparous	334	1011.78	344.66	798.12	1000.24	1183.11	0.730
Primiparous	229	1027.89	338.26	806.99	982.33	1235.26	
Age <35 years	205	1044.98	376.72	803.74	994.68	1261.10	0.468
Age ≥35 years	358	1003.07	319.75	800.90	992.97	1176.04	
Prepregnancy BMI <25kg/m²	343	1051.29	342.50	833.98	1031.56	1248.14	**0.002**
Prepregnancy BMI ≥25 kg/m²	220	966.95	335.21	738.63	921.78	1134.76	
Smoking at recruitment—No	527	1015.94	340.14	796.21	994.36	1207.57	0.612
Smoking at recruitment—Yes	36	1053.31	369.45	849.25	948.26	1224.86	
Higher education—No	175	1030.44	361.58	786.98	1031.02	1237.01	0.721
Higher education—Yes	293	1023.05	334.68	827.71	972.50	1199.15	
Village—No	402	1015.55	340.66	804.74	994.15	1207.57	0.910
Village—Yes	160	1027.58	345.65	788.74	994.48	1214.02	
Lower financial status ***	83	933.87	319.38	750.21	921.90	1137.20	**0.001**
Higher financial status	131	1106.05	337.95	856.61	1063.21	1281.05	
**ZINC (Zn) ***							
Multiparous	334	603.69	84.32	546.78	598.03	653.418	**0.007**
Primiparous	229	640.08	209.67	556.04	623.00	676.42	
Age <35 years	205	626.08	212.83	538.12	607.62	667.01	0.862
Age ≥35 years	358	614.15	96.32	553.29	606.14	660.20	
Prepregnancy BMI <25kg/m²	343	611.65	92.16	550.37	606.61	660.20	0.893
Prepregnancy BMI ≥25 kg/m²	220	629.17	209.65	547.97	606.60	663.57	
Smoking at recruitment—No	527	618.24	152.86	548.04	605.54	663.89	0.353
Smoking at recruitment —Yes	36	622.20	89.34	570.19	627.79	657.91	
Higher education—No	175	640.13	227.81	556.04	610.48	674.72	0.197
Higher education—Yes	293	606.79	87.16	543.31	604.93	656.25	
Village—No	402	614.35	102.14	550.36	607.64	661.26	0.767
Village—Yes	160	629.20	229.31	547.60	603.34	669.09	
Lower financial status	83	641.96	311.84	547.92	606.73	651.05	0.610
Higher financial status	131	614.10	92.51	548.39	607.83	654.15	
**COPPER (Cu) ***							
Multiparous	334	1750.18	293.38	1555.11	1737.13	1938.79	0.065
Primiparous	229	1715.10	356.98	1481.43	1687.54	1904.89	
Age <35 years	205	1733.78	335.20	1525.95	1732.37	1905.78	0.652
Age ≥35 years	358	1737.13	312.94	1543.09	1722.71	1930.14	

Prepregnancy BMI <25kg/m²	343	1665.16	273.38	1480.52	1665.40	1832.70	**1.96E-10**
Prepregnancy BMI ≥25 kg/m²	220	1846.21	357.11	1638.64	1807.34	2021.81	
Smoking at recruitment—No	527	1737.08	324.18	1534.20	1729.44	1923.11	0.645
Smoking at recruitment —Yes	36	1718.79	272.24	1530.15	1647.12	1921.34	
Higher education—No	175	1798.29	340.71	1595.28	1779.06	1985.73	**0.001**
Higher education—Yes	293	1699.65	284.66	1522.93	1690.54	1863.26	
Village—No	402	1731.30	331.03	1529.33	1724.51	1930.14	0.516
Village—Yes	160	1748.43	295.50	1543.81	1733.09	1901.93	
Lower financial status ***	83	1799.13	376.59	1556.88	1772.640	1974.01	0.394
Higher financial status	131	1736.00	300.54	1543.92	1732.40	1910.50	

*Microelement concentrations were measured in serum from the 10–14th gestational week; ** *p*-value was obtained using the Mann–Whitney U test and medians were compared ($p < 0.05$ was considered to be significant); ***financial status in the 5-point Likert scale (lower status included 1–2–3th levels and higher status included 4–5th levels); BMI: Prepregnancy body mass index.

Table 2. Full characteristics of microelement levels (medians and ranges) for pregnancy results.

	n	Mean	SD	Q25	Median	Q75	p **
**SELENIUM (Se) ***							
Normotensive controls	443	61.62	8.66	55.78	61.26	65.82	**2.14E-06**
PIH cases	120	57.56	6.54	53.21	57.47	61.62	
Women without GDM	453	60.48	8.05	55.04	60.02	64.62	0.161
GDM cases	110	61.88	9.74	55.79	61.48	65.91	
IUGR—No	550	60.87	8.40	55.38	60.28	65.11	**0.026**
IUGR—Yes	13	55.59	7.90	50.80	54.62	60.86	
Birth weight ≥10th percentile	515	60.86	8.40	55.49	60.28	65.02	0.303
Birth weight <10th percentile	48	59.60	8.60	52.15	59.37	65.23	
Birth weight ≤90th percentile	499	60.89	8.64	55.08	60.28	65.18	0.296
Birth weight >90th percentile	64	59.66	6.34	55.26	59.34	62.86	
Birth ≥34th week	546	60.88	8.39	55.38	60.28	65.05	**0.043**
Preterm birth <34th week	17	56.71	8.59	51.50	55.30	61.39	
Birth ≥37th week	516	60.91	8.43	55.44	60.30	65.03	0.148
Preterm birth <37th week	47	58.97	8.20	53.34	57.97	65.91	
**IRON (Fe) ***							
Normotensive controls	443	1037.15	341.97	828.96	1006.77	1216.58	**0.014**
PIH cases	120	948.86	333.73	713.34	908.27	1168.33	
Women without GDM	453	1031.52	347.36	823.20	1010.09	1223.09	**0.039**
GDM cases	110	964.03	313.88	751.39	938.32	1127.22	
IUGR—No	550	1020.89	343.14	801.46	994.86	1211.47	0.316
IUGR—Yes	13	910.08	271.15	795.34	938.73	1051.70	
Birth weight ≥10th percentile	515	1022.90	345.31	801.46	995.04	1221.88	0.374
Birth weight <10th percentile	48	969.28	301.28	791.16	932.88	1117.62	
Birth weight ≤90th percentile	499	1024.00	347.23	803.74	995.04	1211.47	0.361
Birth weight >90th percentile	64	974.11	295.33	775.72	922.99	1153.14	
Birth ≥34th week	546	1021.87	343.43	803.74	994.15	1211.47	0.186
Preterm birth <34th week	17	904.82	270.51	699.18	824.42	1104.54	
Birth ≥37th week	516	1026.80	349.09	801.74	994.52	1230.29	0.091
Preterm birth <37th week	47	925.40	232.10	780.91	924.08	1089.67	
**ZINC (Zn) ***							
Normotensive controls	443	620.75	162.28	548.04	606.42	664.99	0.976
PIH cases	120	610.16	87.95	552.91	607.64	659.35	
Women without GDM	453	615.90	158.54	543.31	605.50	659.03	0.084
GDM cases	110	629.20	104.66	568.73	620.77	680.91	
IUGR—No	550	618.06	151.00	548.04	606.45	661.26	0.159
IUGR—Yes	13	636.82	64.06	599.83	635.49	678.88	
Birth weight ≥10th percentile	515	617.32	153.54	547.90	605.54	661.03	0.143
Birth weight <10th percentile	48	631.09	97.19	574.25	628.34	679.32	
Birth weight ≤90th percentile	499	618.81	155.99	547.92	606.61	662.89	0.903
Birth weight >90th percentile	64	616.04	84.85	556.57	607.07	660.04	
Birth ≥34th week	546	619.09	151.14	548.54	607.07	663.89	0.388
Preterm birth <34th week	17	599.35	85.06	553.04	577.96	634.03	
Birth ≥37th week	516	619.75	154.52	547.98	607.07	664.81	0.561
Preterm birth <37th week	47	604.76	75.82	555.54	603.08	642.93	
**COPPER (Cu) ***							
Normotensive controls	443	1745.78	326.15	1534.200	1733.92	1931.60	0.235
PIH cases	120	1699.48	299.35	1528.42	1671.98	1910.50	
Women without GDM	453	1720.77	310.21	1529.56	1717.80	1912.35	0.061
GDM cases	110	1798.27	356.53	1563.33	1786.64	1964.95	
IUGR—No	550	1739.66	319.93	1535.51	1729.41	1930.14	0.113
IUGR—Yes	13	1577.29	336.24	1291.08	1655.65	1750.91	
Birth weight ≥10th percentile	515	1743.77	324.46	1535.51	1733.92	1932.43	**0.031**
Birth weight <10th percentile	48	1651.58	268.92	1496.25	1627.53	1778.93	
Birth weight ≤90th percentile	499	1736.43	323.61	1529.99	1729.39	1923.11	0.853

Birth weight >90th percentile	64	1731.86	301.55	1584.95	1710.01	1929.49	
Birth ≥34th week	546	1732.91	318.39	1532.84	1722.35	1918.57	0.188
Preterm birth <34th week	17	1832.31	393.00	1546.52	1877.77	2130.67	
Birth ≥37th week	516	1734.47	319.89	1532.83	1721.72	1926.20	0.556
Preterm birth <37th week	47	1751.74	335.29	1541.95	1748.77	1912.79	

* Microelement concentrations were measured in serum from the 10–14th gestational week; ** *p*-value was obtained using the nonparametric Mann–Whitney U test and medians were compared ($p < 0.05$ was considered to be significant; PIH: Pregnancy-induced hypertension (105 cases of gestational hypertension and 15 cases of preeclampsia); GDM: Gestational diabetes mellitus (90 cases with dietary modification (GDM-1) and 20 cases with additional insulin therapy (GDM-2)); IUGR: Intrauterine growth restriction.

Table 3. Adjusted relationships between pregnancy results and microelements in the multivariable linear regression.

	Multivariable linear regression			
	**Beta** **	**β** ***	**p** ** **	**R^2** ** ***
**SELENIUM (Se)**				
Pregnancy-induced hypertension (PIH)	**-1.824**	**- 0.178**	**0.0002**	0.085
Isolated gestational hypertension (GH)	**-1.885**	**-0.175**	**0.0002**	0.084
Preeclampsia (PE)	-0.345	-0.013	0.752	0.061
Intrauterine growth restriction (IUGR)	**-5.310**	**-0.095**	**0.022**	0.069
Gestational diabetes mellitus (GDM)	0.773	0.073	0.082	0.066
GDM-1	0.927	0.081	0.053	0.067
GDM-2	-0.118	-0.005	0.902	0.061
Gestational age at birth #	**0.020**	**0.083**	**0.044**	0.113
**IRON (Fe)**				
Pregnancy-induced hypertension (PIH)	-36.466	-0.087	0.068	0.040
Isolated gestational hypertension (GH)	-17.918	-0.041	0.387	0.036
Preeclampsia	**-100.153**	**-0.094**	**0.026**	0.043
Intrauterine growth restriction (IUGR)	-112.247	-0.049	0.240	0.037
Gestational diabetes mellitus (GDM)	-21.883	-0.051	0.233	0.037
GDM-1	-23.770	-0.051	0.229	0.037
GDM-2	-6.461	-0.007	0.869	0.035
Gestational age at birth #	0.0004	0.074	0.083	0.033
**ZINC (Zn)**				
Pregnancy-induced hypertension (PIH)	-14.483	-0.079	0.099	0.028
Isolated gestational hypertension (GH)	-11.505	-0.060	0.207	0.026
Preeclampsia (PE)	-19.064	-0.041	0.336	0.024
Intrauterine growth restriction (IUGR)	18.141	0.018	0.666	0.023
Gestational diabetes mellitus (GDM)	5.115	0.027	0.526	0.024
GDM-1	5.049	0.025	0.562	0.023
GDM-2	3.512	0.009	0.839	0.023
Gestational age at birth #	-0.0003	-0.021	0.612	0.029
**COPPER (Cu)**				
Pregnancy-induced hypertension (PIH)	**-38.487**	**-0.098**	**0.030**	0.142
Isolated gestational hypertension (GH)	-34.348	-0.083	0.062	0.140
Preeclampsia (PE)	-32.837	-0.033	0.412	0.136
Intrauterine growth restriction (IUGR)	-148.820	-0.070	0.080	0.139
Gestational diabetes mellitus (GDM)	27.910	0.069	0.087	0.139
GDM-1	33.454	0.076	**0.057**	0.140
GDM-2	-4.166	-0.005	0.905	0.135
Gestational age at birth #	-0.00003	0.005	0.911	0.028

Relationships between IUGR, PIH, and GDM, and the microelements were calculated after adjusting for age, prepregnancy BMI, gestational age at recruitment, smoking, parity, prior PIH, assisted reproductive technology; # the impact of the microelements on gestational age at recruitment was calculated after adjusting for preeclampsia, delivery by caesarean section, maternal age, prepregnancy BMI, gestational age at recruitment, smoking, parity and fetal sex (the results were not sustained in models with PROM: Premature rupture of membranes); * Beta: Unstandardized regression coefficient; ** β: Standardized regression coefficient; *** $p < 0.05$ was considered to be significant; ** ** R^2: Coefficient of determination; pregnancy-induced hypertension (PIH) included 105 cases of gestational hypertension and 15 cases of preeclampsia; gestational diabetes mellitus (GDM) included 90 cases with dietary modification (GDM-1) and 20 cases with additional insulin therapy (GDM-2).

Table 4. Short characteristics of mothers in the control and case groups (for PIH and preterm birth).

	Controls	Cases	
**Characteristics ****	**Normotensives (n = 443)**	**PIH * (n = 120)**	**p *****
Maternal age ≥35 years	279 (63.0%)	79 (65.8%)	0.564
Prepregnancy BMI ≥ 30 kg/m²	49 (11.1%)	34 (28.3%)	<0.0001
Smokers (at recruitment)	20 (4.5%)	16 (13.3%)	0.0005
Prior PE	1 (0.2%)	3 (2.5%)	0.032
Prior PIH (GH and PE)	3 (0.7%)	13 (10.8%)	<0.0001
ART	19 (4.3%)	11 (9.2%)	0.035
Use of Aspirin in pregnancy	7 (1.6%)	4 (3.3%)	0.259
Multivitamins in II–III trimester **	215 (48.5%)	49 (40.8%)	0.134
Lower education level (for available data)#	127 (34.9%)	48 (46.1%)	0.036
Higher financial status (for available data) ##	99 (65.6%)	32 (50.8%)	0.043
Systolic blood pressure (mmHg) ** **	106.7 (11.3)	158.6 (18.2)	<0.001
Diastolic blood pressure (mmHg) ** **	66.0 (8.9)	100.3 (10.5)	<0.001
Birth weight <10th percentile	26 (5.87%)	22 (18.3%)	<0.001
**Characteristics ****	**Delivery ≥37th week (n = 516)**	**Delivery <37th week (n = 47)**	**p *****
Maternal age ≥35 years	321 (62.2%)	37 (78.7%)	0.024
Prepregnancy BMI ≥ 30 kg/m²	72 (14.0%)	11 (23.4%)	0.080
Smokers (at recruitment)	33 (6.4%)	3 (6.4%)	1.000
Multivitamins in II–III trimester **	241 (46.7%)	23 (48.9%)	0.769
Lower education level (for available data)#	158 (36.7%)	17 (44.7%)	0.329
Higher financial status (for available data) ##	125 (62.5%)	2 (100%)	0.531
Preeclampsia	9 (1.7%)	6 (12.8%)	<0.0007
PROM	42 (8.1%)	10 (21.3%)	0.007
Caesarean section	207 (40.1%)	31 (66.0%)	0.0006
Urinary tract infection	85 (16.5%)	11 (23.4%)	0.226
Birth weight <10th percentile	41 (8.0%)	7 (14.9%)	<0.001

*PIH: Pregnancy-induced hypertension (included 105 cases of gestational hypertension (GH) and 15 cases of preeclampsia (PE)); ** parity was insignificantly associated with PIH and preterm birth; *** the Pearson chi-square test was used for categorical variables comparisons (p-value < 0.05 was considered to be significant); ** ** blood pressure after leaving the postpartum ward; # education other than higher; ## financial status in the 5-point Likert scale (higher status included 4–5th levels); BMI: Body mass index; ART: Assisted reproductive technology; PROM: Premature rupture of membranes.
